# A unique presentation of pulmonary disease in advanced systemic mastocytosis, proven by the presence of mast cells in bronchoalveolar lavage: a case report

**DOI:** 10.1186/s13256-016-1066-5

**Published:** 2016-10-13

**Authors:** Maud A. W. Hermans, Annemiek Broijl, Paul L. A. van Daele

**Affiliations:** 1 Internal Medicine Department, Section of Allergy and Clinical Immunology, Erasmus MC, P.O. Box 2040, 3000 CA Rotterdam, The Netherlands; 2 Internal Medicine Department, Section of Hematology, Erasmus MC, P.O. Box 2040, 3000 CA Rotterdam, The Netherlands

**Keywords:** Mastocytosis, pulmonary, Bronchoalveolar lavage, Mast cells, Case report

## Abstract

**Background:**

Systemic mastocytosis is a rare myeloproliferative disease characterized by the uncontrolled proliferation of aberrant mast cells. It has varying clinical manifestations. For unknown reasons, pulmonary localization of mastocytosis is extremely rare.

**Case presentation:**

In this report, we describe a case of a young Caucasian female with systemic mastocytosis who had an associated hematological non-mast-cell lineage disease with pulmonary interstitial disease directly related to her mastocytosis. The diagnosis was proven by the presence of mast cells in bronchoalveolar lavage. The treatment of her associated hematological disease (myelofibrosis with myelodysplasia) was hampered by rapidly declining pulmonary function and progressive organ dysfunction due to aggressive systemic mastocytosis. She died approximately 1 year after the diagnosis.

**Conclusions:**

To our knowledge, this is the first case in which mast cells were detected in bronchoalveolar lavage. Moreover, to date, only two other cases of pulmonary interstitial disease due to mastocytosis have been published. Juggling therapies for systemic mastocytosis and myelofibrosis is very difficult; however, aggressive therapy for both diseases is essential to give these patients a chance to survive.

## Background

Systemic mastocytosis (SM) is a myeloproliferative disease characterized by uncontrolled proliferation of aberrant mast cells. According to the World Health Organization criteria, different subtypes with different clinical phenotypes and prognoses are identified [1]. When SM coincides with an associated hematological non-mast-cell lineage disease, mostly of myeloid origin, it is called *SM-AHNMD*. To date, there are few effective treatment options for advanced SM. In most patients with SM-AHNMD, only the associated hematological disease is treated. However, conventional myeloablative chemotherapy is not effective in reducing mast cell numbers. This can cause a difficult dilemma of Scylla and Charybdis when SM-AHNMD coincides with aggressive SM causing organ dysfunction.

## Case presentation

The patient, a 26-year-old Caucasian woman, presented to our hematology department after a routine laboratory investigation revealed normocytic anemia with increased myeloid precursor cells, including 5 % blasts. At that time, the patient had no other clinical symptoms aside from a persisting open wound on her lower leg, which was the reason for conducting the first laboratory tests. A bone marrow examination was performed to identify the cause of her anemia. Aspiration of bone marrow was invariably impossible throughout the treatment because of severe myelofibrosis. Therefore, only bone marrow core biopsies were available, which showed a highly hypercellular marrow with an excess of myeloid precursor cells (5 % CD34^+^ and 17 % CD117^+^). There were signs of dysplasia in all cell lines. Furthermore, increased reticulin was seen corresponding to myelofibrosis grade 3. Molecular testing revealed a 47,XX+8 karyogram with a weakly detectable *JAK2 V617F* mutation. A diagnosis of myeloproliferative neoplasia (MPN) with aspects of myelodysplastic syndrome (MDS) was made. On the basis of a high Cervantes score [2], an up-front hematopoietic stem cell transplant (HSCT) was planned, but then postponed because of liver enzyme abnormalities. Analysis revealed only hepatosplenomegaly that was considered secondary to extramedullary hematopoiesis at that time.

**Fig. 1:**
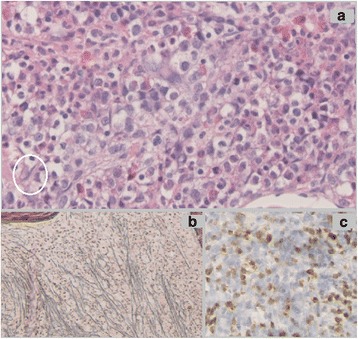
Bone marrow histopathology. **a** A hematoxylin and eosin stain of the second bone marrow core biopsy showed a highly hypercellular marrow with multiple spindle-shaped mast cells (example within the *white oval*). **b** Gömöri stain reveals extensive reticulin fibers, correlating with myelofibrosis grade 3. **c** Tryptase stain shows diffuse infiltration of mast cells. (Published with consent from the Pathology Department of Erasmus MC, Rotterdam.)

**Fig. 2:**
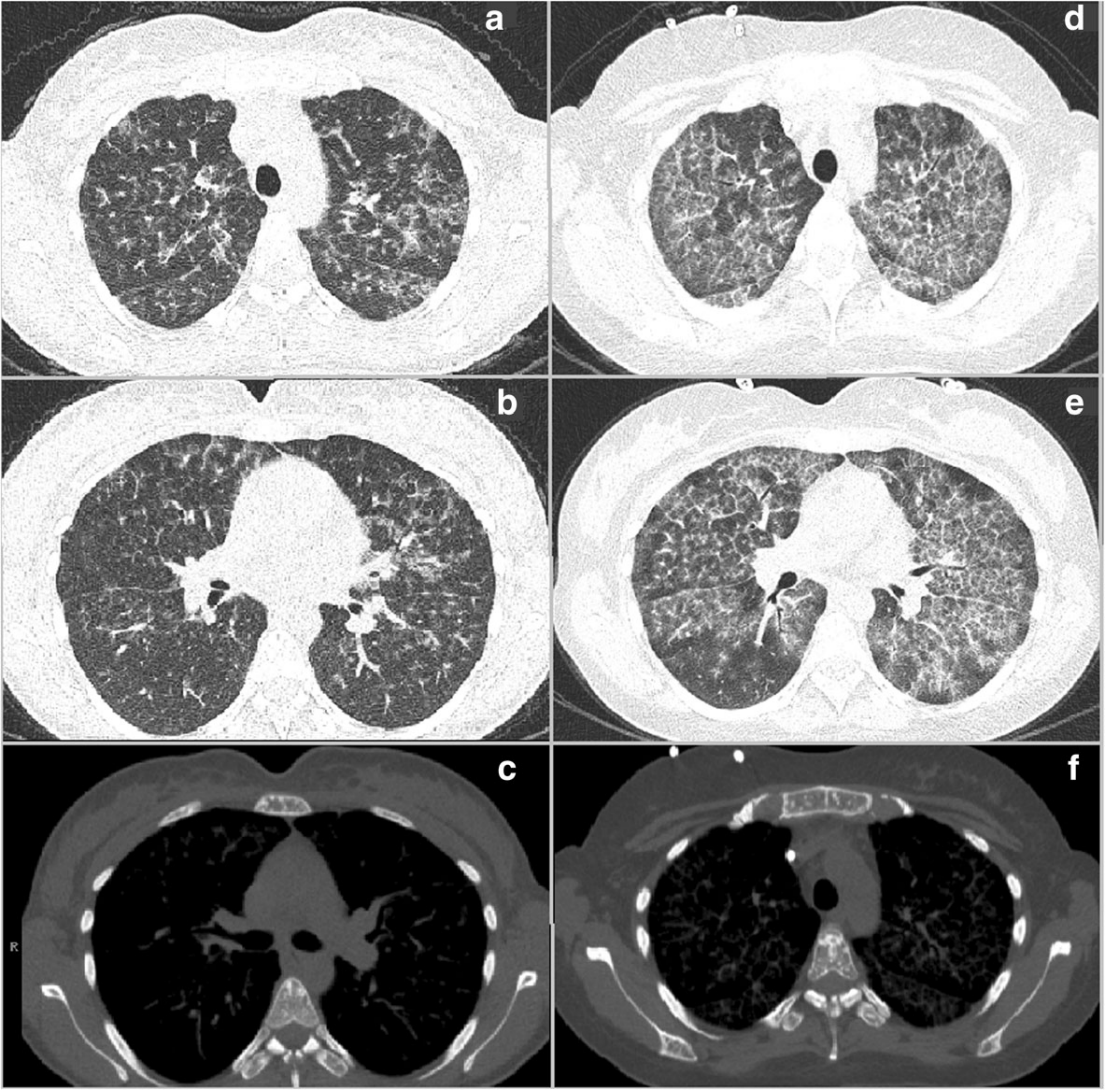
Computed tomographic images obtained at diagnosis (**a**-**c**) and after 4 months (**d**-**f**). **a** and **b** The first computed tomograms of the chest show a ground-glass aspect that is most pronounced in the apical areas with a diffuse reticular aspect and widening of interlobular septa. At this time, the groundglass was mainly localized around bronchovascular bundles. **c** and **f** The skeleton shows multiple focal sclerotic lesions. **d** and **e** Computed tomographic scans obtained 4 months after the first ones show progressive and diffuse reticular ground-glass lesions

Six months later, a follow-up bone marrow examination showed an unexpected finding: Approximately 25 % of the marrow was occupied with clusters of spindle-shaped mast cells (Fig. 1). Immunophenotyping was not feasible, because bone marrow aspiration was unsuccessful; however, the c-KIT *D816V* mutation was detectable, and the patient’s serum tryptase level was elevated (194 μg/L). Thus, the diagnosis was shifted to SM-AHNMD. Simultaneously, the patient developed dyspnea, coughing, fatigue, and diffuse abdominal pain. Like most patients with aggressive SM, she had no mast cell mediator-related symptoms such as pruritus, flushing, or diarrhea. The symptoms had an insidious onset and were gradually progressive with additional fever and weight loss, eventually necessitating admission to the hematology ward. She initially had no hypoxemia. Subsequently, computed tomography (CT) of the chest showed a diffuse reticular aspect with areas of ground-glass appearance and widening of interlobular septa (Fig. 2). The patient had mild restriction with a total lung capacity (TLC) of 3.76 L (77 % of predicted), a forced vital capacity (FVC) of 2.97 L (81 % of predicted), and an impaired diffusing capacity of the lung for carbon monoxide (DLCO) of 62 % of predicted with a corrected diffusion capacity for alveolar volume (TL/VA) of 86 %. Bronchoalveolar lavage (BAL) of the right upper and middle lobes was performed to rule out infectious diseases, mainly *Pneumocystis jirovecii*. Again unexpectedly, mast cells were identified by morphological examination of BAL fluid. Furthermore, neutrophilic and eosinophilic granulocytes, small lymphocytes, and alveolar macrophages were seen. Subsequent immunophenotyping revealed that 8 % of the cells had a phenotype matching neoplastic mast cells (CD117^+^/CD34^−^/HLA-DR^−^/CD2^−^/CD25^+^). No other monoclonal cell population was found. Immunophenotyping of peripheral blood showed that merely 0.3 % had a mast cell phenotype, ruling out contamination of the BAL by blood. A lung biopsy was denied by the patient, but the BAL was considered enough evidence for the interstitial pulmonary disease being directly related to mastocytosis.

The patient had SM-AHNMD with pulmonary involvement, hepatosplenomegaly, skeletal lesions, and lymphadenopathy, with associated MPN/MDS. Concurrent with the aforementioned clinical admission because of worsening pulmonary symptoms, it was noted that the peripheral myeloblasts had increased to 19 %, warranting myeloablative chemotherapy. The Eastern Cooperative Oncology Group status of the patient was 2 at that time. She was treated with high-dose cytarabine and idarubicin in cycle 1, and high-dose cytarabine combined with amsacrine in cycle 2. Complete remission of the MDS was achieved after cycle 2, with no blasts identifiable in a follow-up bone marrow examination. On the basis of knowing that conventional myeloablative chemotherapy is not effective for SM, prednisolone 1 mg/kg was added from the start of the first remission induction cycle. This resulted in short improvement of the pulmonary symptoms and a decrease of the serum tryptase level to 46.8 μg/L. However, repeated CT scans showed progressive disease, which was reflected by pulmonary function testing, with a FVC of 2.36 L (65 %), a forced expiratory volume in 1 second of 1.92 L (61 %), and a DLCO of 58 % of predicted. Moreover, tapering of corticosteroids from 60 to 40 mg/day resulted in quick recurrence of pulmonary symptoms. Therefore, dasatinib was started, but this caused unacceptable side effects (headache and anorexia). Moreover, a rise in blast count was seen 2 months after achievement of complete remission, with a blast count of 20 % in bone marrow. Intensive myeloablative chemotherapy was not considered feasible at that time, owing to the patient’s pulmonary disease with a TLC of 2.86 L (58 %), an FVC of 1.78 L (49 %), a DLCO of 26 %, and a TL/VA of 52 %. In a last effort, cladribine was given to palliatively treat the patient’s mastocytosis. Unfortunately, shortly after the first cycle of cladribine, the patient died as a result of severe neutropenic sepsis with mucositis. Her death occurred 5 months after the discovery of both the presence of SM and the pulmonary interstitial disease.

## Discussion

Here, We describe the extraordinary history of a young woman who died as a result of a very rare complication of SM-AHNMD. To our knowledge, only two cases of interstitial lung disease due to SM have been reported [3, 4]. Furthermore, our case is the first in which mast cells were identified in the BAL. In 2000, Schmidt et al. reported a case of a 54-year-old man with indolent SM who developed mild reticular infiltration of both lungs with infiltration of mast cells evidenced by lung biopsy. Their patient’s pulmonary status improved after 6 months of treatment with interferon-α2A [4]. Furthermore, Kelly et al. described a case of a 70-year-old woman with SM-AHNMD who developed increasing dyspnea with a markedly impaired diffusion capacity and a fine reticular interstitial pattern visualized by chest CT. Lung biopsy showed increased mast cells with septal fibrosis [3]. Unfortunately, no details on treatment or follow-up were given for their case.

Despite the fact that mast cells are abundant in the lungs, pulmonary manifestations of SM are rare. Moreover, when pulmonary involvement is present, it often consists of asthma-like bronchial hyperreactivity or intrapulmonary nodules [5, 6]. We can only speculate on the pathogenesis of interstitial pulmonary disease due to SM. The phenotypical differences in pulmonary versus skin mast cells might provide a (partial) explanation for the rarity of actual pulmonary infiltration by mast cells [7]. Moreover, interstitial fibrosis might contribute to the development of interstitial lesions. It is currently widely accepted that mast cells are important players in the field of fibrosis by both activation of fibroblasts by histamine and direct transforming growth factor-β production [8, 9]. This theory is supported by a case reported by Kelly et al., who found fibrosis in a lung biopsy [3]. However, this still does not explain why so few patients with SM develop pulmonary interstitial disease.

In our patient, the diagnosis of SM was probably missed in the first bone marrow biopsy. When clinical suspicion is lacking, aberrant mast cells can be missed easily, especially in such hypercellular marrow (Fig. 1) [10]. Next, when the patient developed interstitial lung lesions, again an accidental finding of mast cells in the BAL with morphological examination gave away the diagnosis of mastocytosis-related pulmonary interstitial disease. This would have been missed without morphological examination. A high index of suspicion is therefore needed to diagnose pulmonary mastocytosis.

The combination of these two aspects of SM-AHNMD leads to a difficult dilemma of Scylla and Charybdis, as there are currently no cytoreductive therapies that can treat both MDS with excessive blast count and SM. In our case, fear for toxicity of combining two types of cytoreductive treatments probably led to undertreatment of the patient’s mastocytosis. The patient then went into a downward spiral in which the pulmonary lesions hampered any further intensive treatment. One could wonder whether the patient’s mastocytosis should have been treated more aggressively from the moment it was diagnosed, even postponing the treatment of the MPN/MDS. In 2009, Ustun et al. reported a case of an adult patient with aggressive SM with acute myeloid leukemia (AML) in whom dasatinib was added in an early phase of myeloablative therapy, which resulted in long-term remission of both the AML and the mastocytosis [11]. When feasible, HSCT can also induce long-term remission of advanced SM, and up-front HSCT is probably the treatment of choice [12].

## Conclusions

This report describes a very rare case of interstitial lung disease due to aggressive SM in a patient with SM-AHNMD. It is important that awareness is increased for this rare but lethal complication of aggressive SM. Immunophenotyping of BAL cells can serve as an alternative and less invasive method of obtaining a diagnosis in cases where a lung biopsy is not feasible. Timely and aggressive cytoreductive treatment of both the SM and the associated hematological disease is of paramount importance with regard to the survival of these patients.

## Abbreviations

AML: Acute myeloid leukemia
CT: Computed tomography
SM: Systemic mastocytosis
SM-AHNMD: Systemic mastocytosis with associated hematological non-mast-cell lineage disease
MPN: Myeloproliferative neoplasia
MDS: Myelodysplastic syndrome
TLC: Total lung capacity
FVC: Forced vital capacity
DLCO: Diffusing capacity of the lung for carbon monoxide
TL/VA: Corrected diffusion capacity for alveolar volume
HSCT: Hematopoietic stem cell transplant
BAL: Bronchoalveolar lavage
